# Bone Anabolic Effects of Soluble Si: *In Vitro* Studies with Human Mesenchymal Stem Cells and CD14+ Osteoclast Precursors

**DOI:** 10.1155/2016/5653275

**Published:** 2015-12-20

**Authors:** J. Costa-Rodrigues, S. Reis, A. Castro, M. H. Fernandes

**Affiliations:** Faculdade de Medicina Dentária, Universidade do Porto, 4200-393 Porto, Portugal

## Abstract

Silicon (Si) is indispensable for many cellular processes including bone tissue metabolism. In this work, the effects of Si on human osteogenesis and osteoclastogenesis were characterized. Human mesenchymal stem cells (hMSC) and CD14+ stem cells, as osteoblast and osteoclast precursors, were treated with a wide range of Si concentrations, covering the physiological plasma levels. Si promoted a dose-dependent increase in hMSC proliferation, differentiation, and function, at levels similar to the normal basal plasma levels. Additionally, a decrease in the expression of the osteoclastogenic activators M-CSF and RANKL was observed. Also, Si elicited a decrease in osteoclastogenesis, which became significant at higher concentrations, as those observed after meals. Among the intracellular mechanisms studied, an upregulation of MEK and PKC signalling pathways was observed in both cell types. In conclusion, Si appears to have a direct positive effect on human osteogenesis, at basal plasma levels. On the other hand, it also seemed to be an inhibitor of osteoclastogenesis, but at higher concentrations, though yet in the physiological range. Further, an indirect effect of Si on osteoclastogenesis may also occur, through a downregulation of M-CSF and RANKL expression by osteoblasts. Thus, Si may be an important player in bone anabolic regenerative approaches.

## 1. Introduction

Bone metabolism is highly dynamic, requiring multiple cross-talks between different cell types. Among them, there are two that are key players in the process [1, 2]. Osteoblasts, the bone-forming cells that descend from the pluripotent mesenchymal stem cell population, are cuboid cells specialized in the formation of bone extracellular matrix [3]. At a first stage, they secrete osteoid, which corresponds to the nonmineralized bone matrix, which is mainly composed of collagen type 1 fibres. Then, they start to deposit calcium phosphate crystals, in the form of hydroxyapatite [2, 3]. Osteoclasts are bone-resorbing cells that originate from the fusion and further differentiation of their CD14+ stem cell precursors [4, 5]. They are multinucleated cells that promote the dissolution of hydroxyapatite and degradation of bone matrix proteins by means of acidification and secretion of proteases, respectively [5, 6]. Osteoblast-osteoclast reciprocal communications are indispensable for the establishment of an adequate equilibrium between both cellular activities, and, consequently, a proper bone structure and function [7–9].

In addition to being one of the most abundant elements in nature, silicon (Si) is also an indispensable player in many different biochemical processes in the human body. Its predominant physiological soluble form is as silicic acid [10, 11], and it is mainly found in blood and particularly in connective tissues such as cartilage and bone [11, 12], where it acts as an important modulator of local metabolism. The importance of Si in bone metabolism is recognized for years. In the 1970s, it was observed that Si deprivation negatively affected bone development in rats and chicks [13, 14]. Some years later, a positive association between Si intake and bone mineral density in humans was reported [15], and also with a decreased osteoporosis risk [16]. From a structural point of view, it is known that Si is important for the production of bone extracellular matrix, leading to a more effective cross-linking and stabilization of the network of collagen and glycosaminoglycans fibers, being also important for the formation of hydroxyapatite [11, 13, 14]. At the cellular level, it has been suggested that it acts as a positive modulator of osteoblast anabolic activity [17–21], while rendering an opposite effect on osteoclastic cells [10, 22–26]. Since the available information relies mainly on studies with nonhuman cells and also the chemical form (ranging from soluble forms to biomaterials containing Si) and the tested doses of Si are very different among the published reports, it is important to standardize the experimental conditions, in order to gather more detailed information about the real effects of Si on human bone tissue. The present study aims to investigate the effects of the most common Si soluble form, silicic acid, on both primary human osteoblasts and osteoclasts. The tested range of Si concentrations covers the normal plasma basal Si levels (2–10 *μ*M), as well as the increased levels achieved after a meal (~30 *μ*M) [11, 12, 18]. Osteoblast differentiation from mesenchymal stem cells and osteoclast differentiation from CD14+ stem cells were evaluated at both cellular and molecular levels. The involvement of several intracellular signalling pathways on cellular response was also analyzed.

## 2. Materials and Methods

### 2.1. Human Mesenchymal Stem Cell (hMSC) Cultures

Human bone marrow-derived mesenchymal stem cells (hMSC) were purchased from ATCC (PCS-500-012). hMSC were cultured in *α*-Minimum Essential Medium (*α*-MEM) containing 10% foetal bovine serum, 100 IU/mL penicillin, 2.5 *μ*g/mL streptomycin, 2.5 *μ*g/mL amphotericin B, and 50 *μ*g/mL ascorbic acid. After reaching ~70% confluence, adherent cells were enzymatically detached with 0.04% trypsin and 0.025% collagenase and seeded (10^4^ cell/cm^2^) in the same medium as described above. After 24 h incubation, cell cultures were supplemented with 10 mM of *β*-glycerophosphate, as a source of phosphate ions, and 10 nM dexamethasone, as an osteogenic inducer [27], and further treated with silicic acid, as described below.

### 2.2. CD14+ Stem Cell Cultures

CD14+ cells were isolated from blood of healthy male donors at the age of 25–35 years, as described previously [28]. Shortly, after dilution with PBS (1 : 1), blood was applied on top of Ficoll-Paque PREMIUM (GE Healthcare Bio-Sciences) and centrifuged for 30 min at 400 g. Mononuclear cells were collected and washed twice with PBS. Afterwards, CD14+ cells were isolated with the Magnetic Cell Sorting (MACS, Miltenyi Biotec). For that, peripheral blood mononuclear cells were incubated with MACS magnetic microbeads conjugated with a monoclonal mouse antibody anti-human CD14. Samples were added to a column and subjected to a magnetic field. After washing with PBS, CD14+ cells were eluted following magnetic detachment of the column. Cells were seeded at 1.5 × 10^5^ cells/cm^2^ in *α*-MEM supplemented with 30% (V/V) autologous human serum, 2 mM L-glutamine, 100 IU/mL penicillin, 2.5 *μ*g/mL streptomycin, and 2.5 *μ*g/mL amphotericin B. After 24 h incubation, cell cultures were maintained in the same culture medium described above, supplemented with the recombinant osteoclastogenic promoters M-CSF (25 ng/mL) and RANKL (40 ng/mL) [4, 6], and treated with silicic acid, as described below.

### 2.3. Exposure of Cell Cultures to Si and Characterization of Cell Behaviour

hMSC and CD14+ cell cultures, maintained in the absence (control) or presence of silicic acid (0.2–125 *μ*M), were incubated for 21 days at 37°C in a 5% CO_2_ humidified atmosphere. Culture medium was replaced once a week and Si was renewed at each medium change. Cell cultures were characterized for total DNA content, alkaline phosphatase/tartrate-resistant acid phosphatase activity, and apoptosis at days 7, 14, and 21. Representative experimental values related with Si concentrations that caused different effects on bone cells are presented, including the minimal required concentration (25 *μ*M) for Si-related effects in both hMSC and CD14+ cells: more precisely, the maximum stimulatory effect in hMSC cultures and simultaneously the lowest concentration eliciting a significant inhibitory profile in CD14+ cell cultures. Cell cultures treated with this Si concentration were further characterized for gene expression, functional parameters (mineralization/resorbing ability), cellular morphology, and the involvement of different intracellular signalling pathways in cellular response.

### 2.4. Total DNA

DNA quantification of hMSC and CD14+ cell cultures was performed using PicoGreen dsDNA kit (Molecular Probes, Eugene, USA), according to the manufacturer's instructions. Briefly, cells were washed twice with PBS and solubilized with 0.1% (V/V) Triton X-100 for 15 min. Samples were transferred to a black 96-well plate and PicoGreen dye was added to each sample. After 2 minutes of incubation at room temperature, fluorescence was measured at 485/520 nm (excitation/emission) in an ELISA plate reader (Synergy HT; Biotek). Results are expressed as pg of DNA.

### 2.5. Caspase-3 Activity

In order to evaluate if Si was able to modulate cell death by apoptosis, cells maintained in the absence or presence of Si were assessed for caspase-3 activity, an effector caspase involved in both apoptotic pathways (extrinsic and intrinsic). Caspase-3 activity of hMSC and CD14+ cell cultures was performed using EnzChek Caspase-3 Assay Kit #2 (Molecular Probes, Eugene, USA), according to the manufacturer's instructions. Cell cultures, at days 14 and 21, were washed twice with PBS and solubilized with 0.1% (V/V) Triton X-100 for 15 min. Samples were transferred to a black 96-well plate and incubated with Z-DEVD-R110 substrate for 30 minutes at room temperature. After incubation, fluorescence was measured at 485/520 nm (excitation/emission) in an ELISA plate reader (Synergy HT; Biotek). Results obtained were normalized with the corresponding DNA content and presented as a percentage of the response obtained in the control (absence of Si).

### 2.6. Actin Staining

hMSC and CD14+ cell cultures, at day 21, were washed twice with PBS and were fixed with 3.7% paraformaldehyde for 15 minutes at room temperature. Cells were treated with 0.1% (V/V) Triton X-100 for 5 minutes and stained for F-actin with 5 U/mL Alexa Fluor 647-Phalloidin (Invitrogen, Massachusetts, USA). Cultures were observed by confocal laser scanning microscopy (CLSM) with a Leica TCP SP2 AOBS confocal microscope (Leica, Wetzlar, Germany).

### 2.7. Alkaline Phosphatase (ALP) and Tartrate-Resistant Acid Phosphatase (TRAP) Activity

ALP (hMSC cultures) and TRAP (CD14+ cell cultures) activities were quantified by the para-nitrophenylphosphate (*p*NPP) hydrolysis assay. Cell layers were washed twice with PBS and solubilized with 0.1% (V/V) Triton X-100 for 15 min. For ALP activity, hMSC cultures were incubated (1 hour at 37°C) with 12.5 mM* p*NPP in 0.15 M bicarbonate buffer solution, pH 10.3. For TRAP activity, CD14+ cell cultures were incubated (1 hour at 37°C) with 12.5 mM* p*NPP in 0.04 M tartaric acid and 0.09 M citrate (pH 4.8). After incubation, the reaction was stopped with 5 M NaOH, and the absorbance of the samples was measured at 400 nm in an ELISA plate reader (Synergy HT; Biotek). Results were normalized to total DNA content and expressed as nmol/min·pg_DNA-1_.

### 2.8. TRAP+ Multinucleated Cells

CD14+ cell cultures were washed twice with PBS and fixed with 3.7% formaldehyde for 10 min, at room temperature. Then, samples were rinsed with distilled water and stained for TRAP with Acid Phosphatase, Leukocyte (TRAP) kit (Sigma). Cells were incubated with naphthol AS-BI 0.12 mg/mL, in the presence of 6.76 mM tartrate and 0.14 mg/mL Fast Garnet GBC, at 37°C for 1 hour in the dark. Then, cells were washed and stained with hematoxylin. The amount of TRAP+ multinucleated cells in each experimental condition was quantified under light microscopy (Nikon TMS).

### 2.9. Osteoblast and Osteoclast Gene Expression

hMSC and CD14+ cell cultures, maintained in the absence or presence of 25 *μ*M Si, were analyzed by qPCR at day 21. hMSC cultures were characterized for the expression of early osteogenesis markers, namely, the transcription factor Runx2 and the functional osteoblastic genes collagen type 1 (COL1) and ALP, as well as late osteogenesis markers, such as osteocalcin (OC), bone sialoprotein (BSP), and osteopontin (OP) and the osteoclastogenic modulators M-CSF and RANKL [1, 3]. CD14+ cultures were analyzed for the expression of the transcription factors, c-myc and c-src, and the functional osteoclastic genes, TRAP, cathepsin K (CATK), and carbonic anhydrase 2 (CA2) [1, 6]. The genes coding for beta-glucuronidase (GUSB) and proteasome subunit beta type-6 (PSMB6) were used as housekeeping genes. RNA was isolated with RNeasy Mini Kit (Qiagen), according to the manufacturer's instructions. cDNA synthesis was performed with DyNAmo cDNA synthesis kit (Finnzymes) and random hexamers according to the manufacturer's instructions. 2 ng of each cDNA sample was amplified with a DyNAmo Flash SYBR green qPCR kit (Finnzymes) on a Rotor-Gene thermocycler (Qiagen). The annealing temperature used was 55°C and the extension time was 15 seconds. The primers used are listed in Table 1. The values obtained in each condition were normalized with the results obtained for the housekeeping genes in the same experimental condition, and the results were presented as relative expression.

### 2.10. Staining of Phosphate Deposits (Von Kossa Staining)

hMSC cultures were washed twice with PBS and fixed with 1.5% glutaraldehyde in 0.14 M sodium cacodylate buffer for 10 min, at room temperature. Then, cell layers were covered with a 10 mg/mL silver nitrate solution and incubated for 1 h under UV light. Samples were rinsed with deionized water and incubated for 2 min with a 50 mg/mL sodium thiosulphate solution. After being washed with deionized water, phosphate deposits (stained black) were visualized under a phase-contrast optical microscope (Nikon TMS).

### 2.11. Staining of Calcium Deposits (S Alizarin Red Staining)

After being washed twice with PBS, hMSC cultures were fixed for 10 min with 1.5% glutaraldehyde in 0.14 M sodium cacodylate buffer. Samples were covered with 1% S alizarin sodium solution (0.028% in NH_4_OH), for 2 min, and then rinsed with distilled water and acid ethanol (ethanol, 0.01% HCL). Calcium deposits stained red were visualized under a phase-contrast optical microscope (Nikon TMS).

### 2.12. Calcium Phosphate Resorbing Ability

CD14+ cells were cultured for 21 days on BD BioCoat Osteologic Bone Cell Culture Plates (BD Biosciences). Cells were removed by incubation with 6% NaOCl and 5.2% NaCl, according to the manufacturer's instructions. Calcium phosphate layers were visualized under a phase-contrast light microscope and image analysis of the resorbed areas was performed with ImageJ 1.41 software.

### 2.13. Intracellular Signalling Mechanisms

Osteoblastic and osteoclastic responses to Si were assessed by means of the involvement of several intracellular signalling pathways known to be important for osteoblast and osteoclast development [1, 3, 6]. For that, hMSC and CD14+ cell cultures were treated with the commercial signalling pathway inhibitors throughout the culture time. The tested pathways were MEK (inhibitor U0126, 1 *μ*M), NFkB (inhibitor PDTC, 10 *μ*M), PKC (inhibitor GO6983, 5 *μ*M), JNK (inhibitor SP600125, 10 *μ*M), p38 (inhibitor SB202190, 5 *μ*M), MAPKK (inhibitor PD98,059, 10 *μ*M), and PGE2 production (inhibitor indomethacin, 1 *μ*M). At days 14 and 21, cultures were assessed for ALP (hMSC) and TRAP (CD14+) activities.

### 2.14. Statistical Analysis

Data were obtained from three separate experiments, each one performed in triplicate. Results are expressed as mean ± standard deviation. Groups of data were evaluated using a two-way analysis of variance (ANOVA) and no significant differences in the pattern of the cell behaviour were found. The statistical significance of cell response compared to the controls was analyzed using a two-tailed Student's* t*-test and corrected by Bonferroni's method. Values of *P* < 0.05 were considered significant.

## 3. Results

### 3.1. Effects of Si on Osteoblastic Cells

**Figure 1:**
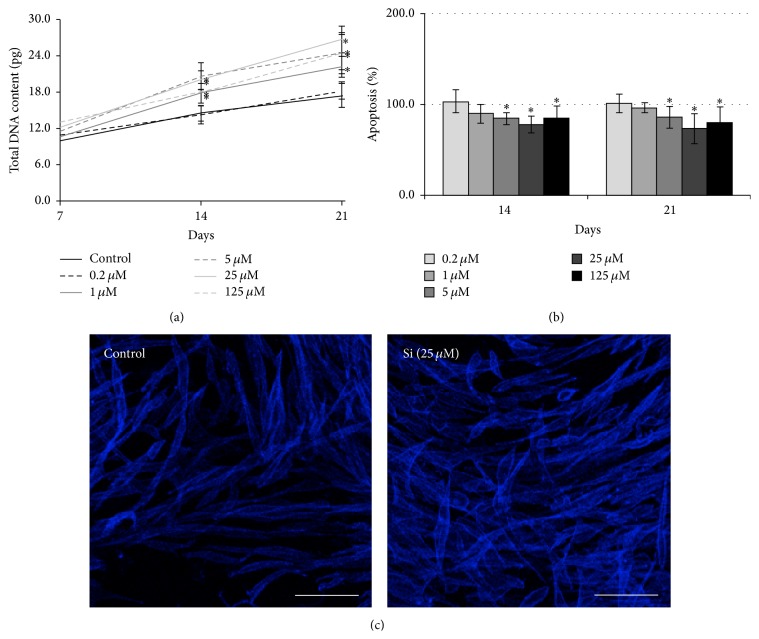
Effects of Si on cell density, apoptosis, and morphology of hMSC cultures. (a) Cellular density, determined by total DNA content; (b) apoptosis, determined by caspase-3 activity; (c) actin staining and visualization under confocal laser scanning microscopy. ^*∗*^Significantly different than the control. White bars represent 150 *μ*m.

hMSC were cultured in an osteogenic culture medium, containing different Si concentrations, ranging from 0.2 to 125 *μ*M (Figure 1). Cell cultures performed in the absence of Si were used as control.

Total DNA content of control cultures increased with the increase in culture period (Figure 1(a)). The presence of Si caused a dose-dependent increase in the DNA content, which became statistically significant for concentrations ≥ 1 *μ*M. The maximum response was achieved in the presence of 25 *μ*M (~53% higher than the control).

Regarding the effects on apoptosis, a negative effect dependent on the concentration of Si was observed (Figure 1(b)). That means, compared to the control, cells treated with Si (≥5 *μ*M) presented a lower caspase-3 activity (~26% lower in the presence of Si 25 *μ*M). No significant differences were found between day 14 and day 21.

Cells (either in the control or treated with Si) exhibited a wide distribution in the culture plates, with an expected morphology (Figure 1(c)). In the presence of Si, a higher cell number was observed, with a more evident nodular organization of the cell layer. Actin filaments were well organized, with a homogeneous cytoplasmatic distribution.

**Figure 2:**
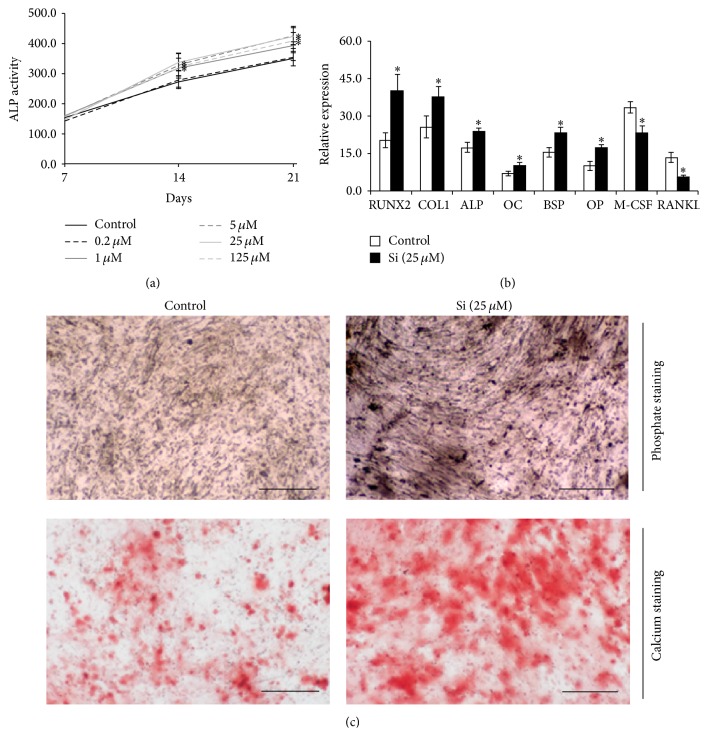
Effects of Si on the differentiation and function of hMSC. (a) ALP activity, determined by* p*NPP hydrolysis assay. (b) qPCR analysis of cell cultures maintained in the absence (control) or presence of Si (25 *μ*M); (c) presence of phosphate and calcium deposits in cell layers, by von Kossa and alizarin red staining, respectively. ^*∗*^Significantly different than the control. Black bars represent 300 *μ*m.

ALP activity increased at the different culture time points (Figure 2(a)). Compared to the control, cell cultures treated with Si (≥5 *μ*M) exhibited a higher enzyme activity, being ~22% higher for Si concentrations of 5 and 25 *μ*M.

After establishing a dose-response curve for Si on osteoblastic cells, the concentration that elicited a maximum response (25 *μ*M) was chosen to evaluate the effects of Si on osteoblast gene expression and on the extracellular matrix production (Figures 2(b) and 2(c)).

Either in the absence or in the presence of Si, cells exhibited a positive expression of all the tested genes (Figure 2(b)). Nevertheless, the presence of Si caused a significant increase in the expression of either early osteogenesis markers RUNX2, COL1, and ALP (resp., ~96, 49, and 34%) or late markers OC, BSP, and OP (~44%, ~50%, and ~71%); on the other hand, a downregulation in the expression of the osteoclastogenic modulators M-CSF and RANKL was observed (~30% and ~59%, resp.).

hMSC cultures maintained in the absence or presence of Si were analyzed for their mineralization ability, by means of phosphate and calcium staining (Figure 2(c)). Si significantly increased the amount of the deposits of both ions, with a uniform distribution throughout all the cell layer.

### 3.2. Effects of Si on Osteoclastic Cells

**Figure 3:**
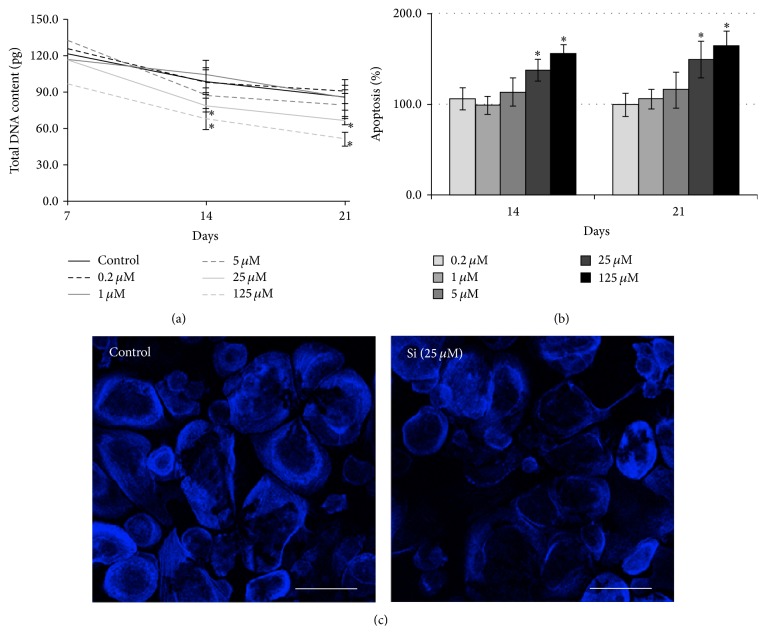
Effects of Si on cell density, apoptosis, and morphology of CD14+ cell cultures. (a) Cellular density, determined by total DNA content; (b) apoptosis, determined by caspase-3 activity; (c) actin staining and visualization under confocal laser scanning microscopy. ^*∗*^Significantly different than the control. White bars represent 150 *μ*m.

Cell cultures of CD14+ were maintained in an osteoclastogenic medium, in the absence (control) or presence of 0.2–125 *μ*M Si (Figure 3).

All the cell cultures exhibited a time-dependent decrease in total DNA content (Figure 3(a)). Supplementation with Si concentrations ≥25 *μ*M elicited a significant dose-dependent decrease of cell response (~23% lower than the control).

The presence of Si at low concentrations did not significantly affect apoptosis (Figure 3(b)). However, concentrations ≥ 25 *μ*M caused a significant dose-dependent increase in the values.

The presence of round-shaped cells displaying actin rings in both tested conditions was observed (Figure 3(c)). The amount of those cells was lower in cell cultures supplemented with Si. Furthermore, actin rings appeared less organized in that experimental condition, with a significant lower thickness than the rings observed in the control.

**Figure 4:**
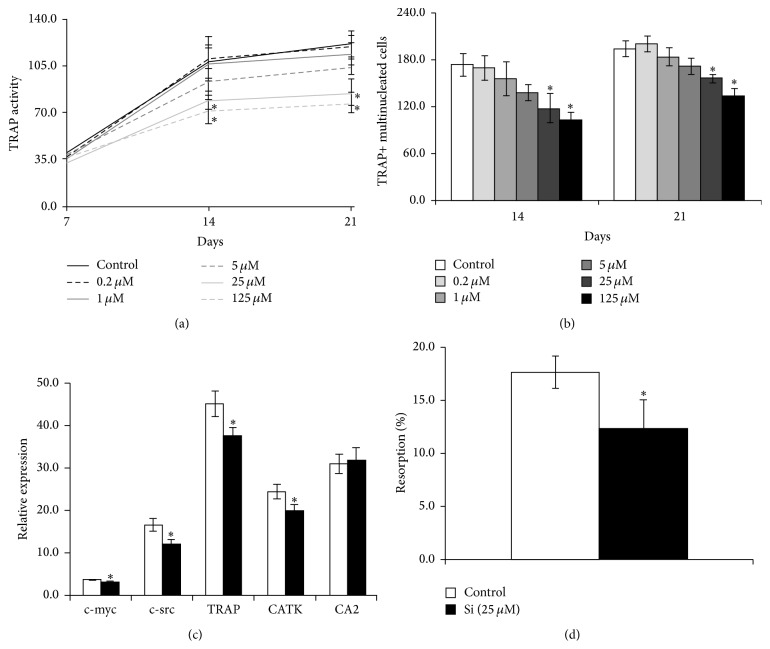
Effects of Si on the differentiation and function of CD14+ cells. (a) TRAP activity, determined by* p*NPP hydrolysis assay; (b) number of TRAP+ multinucleated cells, determined by TRAP and hematoxylin staining; (c) qPCR analysis of cell cultures maintained in the absence (control) or presence of (25 *μ*M) Si; (d) calcium phosphate resorbing activity of cell cultures. ^*∗*^Significantly different than the control.

TRAP activity increased with the culture period, being more pronounced between days 7 and 14 (Figure 4(a)). Cell cultures treated with Si exhibited a dose-dependent decrease of TRAP activity, which became statistically significant for concentrations ≥ 25 *μ*M (~30% lower than the control).

The amount of TRAP+ multinucleated cells in the different experimental conditions (Figure 4(b)) followed a similar pattern of response compared to that observed for TRAP activity.

After determining the pattern of response of osteoclastic cells to different Si concentrations, CD14+ cell cultures were performed in the absence or presence of 25 *μ*M Si, which corresponds to the lowest concentration that elicited a significant inhibitory effect in osteoclast development. Cell cultures were further characterized for osteoclast gene expression and resorbing ability (Figures 4(c) and 4(d)).

All the tested genes were expressed in both conditions (Figure 4(c)). The presence of Si caused a significant decrease in the expression of all genes (~13–28%), except in the case of CA2, which was not significantly affected by the chemical element.

Regarding the resorbing ability, cell cultures treated with Si exhibited a decrease in cell response, being about ~30% lower than the control (Figure 4(d)).

### 3.3. Modulation of Intracellular Signalling Pathways by Si

**Figure 5:**
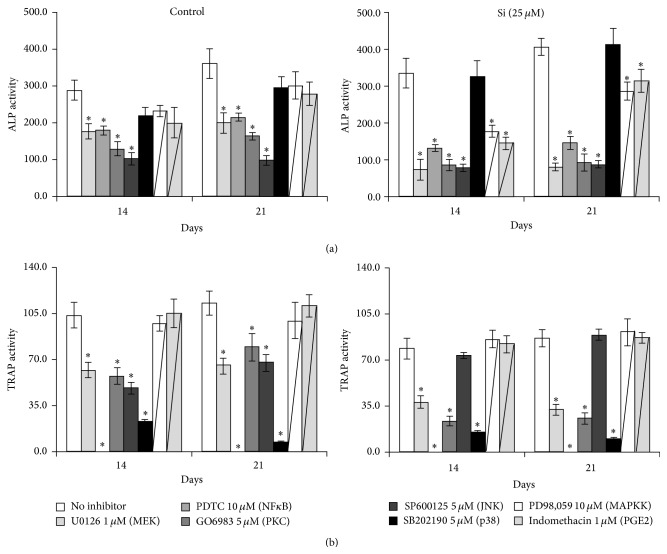
Modulation of intracellular signalling pathways by Si. Cell cultures were treated with several inhibitors and assessed for ALP activity (hMSC cultures, panel (a)) or TRAP activity (CD14+ cell cultures, panel (b)). ^*∗*^Significantly different from the absence of inhibitors. Black arrows represent the main differences observed in Si-treated cultures, compared to the corresponding negative controls (absence of Si).

hMSC and CD14+ cell cultures, performed either in the absence or in the presence of 25 *μ*M Si, were characterized for the involvement of several signalling pathways important for osteoblastogenesis and osteoclastogenesis, respectively (Figure 5).

In control conditions, hMSC cultures revealed a decrease in ALP activity in the presence of U0126 (~45%), PDTC (~40%), GO6983 (~55%), and SP600125 (~72%) (Figure 5(a)). The remaining inhibitors (SB202190, PD98,059, and indomethacin) did not significantly affect cell response at day 21. Supplementation with Si caused an increase in the inhibitory profile of several tested inhibitors, particularly U0126 (~80%), GO6983 (~76%), PD98,059 (~29%), and indomethacin (~22%).

CD14+ cell cultures maintained in the absence of Si exhibited a significant inhibition of TRAP activity when treated with U0126 (~42%), GO6983 (~29%), SP600125 (~40%), and SB202190 (~94%) (Figure 5(b)); PDTC totally abolished TRAP activity. In the presence of Si, the inhibition elicited by U0126 (~63%) and GO6983 (~70%) was potentiated, while SP600125 turned to have no effect on cell behaviour.

## 4. Discussion

Silicon is a very abundant chemical element in nature. In human body, it is present in low amounts, though it is recognized as crucial for several physiological processes, including bone and cartilage metabolism [11, 12, 16]. In line with this, in human body Si is mainly located in bone tissue [11], and* in vivo*, it was reported that Si increases human bone mineral density and decreases the risk of osteoporosis [11, 15, 16, 29]. The present study aimed to analyze the* in vitro* cellular and molecular effects of Si on human osteogenesis and osteoclastogenesis. For that, mesenchymal stem cells and CD14+ stem cells were used as osteoblastic and osteoclastic precursors, respectively.

The chemical form of Si chosen to be tested was silicic acid, since this represents the main form of Si in drinking water and in the food after digestion, and it displays a high bioavailability in humans (>50%), being the main form that is absorbed in the intestine [30, 31]. Also, Si concentration in the human body is highly variable, ranging from plasma basal levels of 2–10 *μ*M to increased levels achieved after a meal (~30 *μ*M), or even higher values in muscle and lungs [12, 16, 18], although values higher than 50 *μ*M may be considered as supraphysiological [18]. The tested concentration range was selected in order to cover all the physiological level range of Si, achieved before, between, or after meals.

Si was able to promote a significant increase in osteoblast proliferation, at basal levels found in plasma between meals; this effect may be related, at least partly, by a decrease in apoptosis. Also, a nodular organization of cell layer, which is a distinctive osteoblastic behaviour [3], and a well-defined cytoskeleton composed of a complex network of actin filaments were more evident in cell cultures treated with Si. In line with this, a stimulation of osteoblast differentiation and function was observed. Si was able to elicit an increase in ALP activity, which suggests a high osteogenic differentiation degree of hMSC. This was supported by a significant stimulation in the expression of RUNX2, which codes for a key transcription factor for osteogenesis [1, 3], as well as other important osteoblast genes involved in the first steps of osteoblast development (COL1 and ALP) and in late osteogenesis events (OC, BSP, and OP) [1, 3], suggesting an increase in the formation of fully differentiated osteoblasts; in line with this, a significant increase in the production of calcium phosphate-containing extracellular matrix was also observed. This stimulation is in agreement with one of the main proposed effects of Si on bone tissue, which is the positive modulation of extracellular matrix production [11, 12]. Also, it is important to highlight the decrease in the expression of the osteoclastogenic activators M-CSF and RANKL. Since osteoblasts are key modulators of osteoclastogenesis, this result suggests an indirect mode of action of Si on osteoclast development, via osteoblasts [1, 2].

The effects of different forms of Si on osteoblasts were previously analyzed. In studies conducted with a human osteosarcoma cell line, SaOS-2, or with the mouse osteoblastic cell line, MC3T3-E1, or bone marrow cells, it was observed that biosilica promoted osteogenesis, by increasing the expression of osteoprotegerin and bone morphogenetic protein 2, as well as the biomineralization [17–21]. In another study, conducted with MC3T3-E1 cells, sodium metasilicate (0–1000 *μ*M) promoted cell proliferation and differentiation [17], although collagen expression was only increased in the presence of 50 *μ*M metasilicate, and differences in the expression of RANKL were not observed. In a work conducted with the human osteosarcoma MG63 cell line, it was observed that Si concentrations of 2 and 4 mM stimulated cell proliferation and function, and concentrations higher than 4 mM elicited an opposite effect, by stimulating apoptosis [32]. In a coculture of mouse bone marrow cells and RAW 264.7 cells, amorphous silica from collagen scaffolds elicited a decrease in RANKL expression, as well as an upregulation of osteogenesis [33]. Also, silicic acid, at 10–20 *μ*M, elicited an increased osteogenic response and especially an upregulation of collagen type 1 expression in MG63 cell line, as well as in human bone marrow cells, though a concentration of 50 *μ*M revealed a marked lower stimulatory effect [18]. Due to the anabolic effects of Si on bone metabolism, extensive research has also been conducted for years in the development of biomaterials containing this element [34–36]. Many different bioceramic or ceramic-glass composites have been developed, with differences in the source of Si, in the formulation, and in the strategies of Si inclusion in the material. In many of them, promising results have been observed in* in vitro* evaluations, with various degrees of stimulation on osteoblast proliferation/differentiation [20, 37–41]. Summing all up, despite the high variability of tested cell types, combined with the difference sources, chemical forms, and doses of Si, it is recognized that Si elicits a stimulatory effect on osteogenesis, which is in agreement with the present results.

Regarding the Si-related effects observed in osteoclastogenesis, Si promoted a decrease in cell density, while an increase in apoptosis was noted, suggesting that Si may promote osteoclast (or its precursors) cell death by upregulating apoptosis. In parallel, a decrease in the reorganization of the actin fibres into actin rings was noted, being also observed that the thickness of the formed rings was also marked lower than in the control. This is an important finding, since actin rings are very important structures for bone resorption [1, 5]. In line with this, it was observed that osteoclastogenesis was decreased by Si, by means of TRAP production and also expression of the osteoclast differentiation and activation factors, c-myc and c-src, respectively [6], and also osteoclast functional genes such as TRAP and CATK. This effect was also observed at the cellular function of osteoclasts, since their ability to resorb a calcium phosphate substrate was decreased by Si. This effect was the opposite of what was obtained for osteoblasts. Moreover, an important finding deserves to be mentioned. The effects on osteoclasts were observed mainly in a concentration range that is higher than the normal Si basal plasma levels, although it is below the maximum levels obtained after meals (30 *μ*M). Although osteoclasts are key players in bone metabolism, the effects of Si in this cell type are scarcely documented, with only a few studies addressing this issue. It was previously described that silicate ions (3–100 mM) did not affect the behaviour of the murine cell line RAW 264.7, when cocultured with SaOS-2 cells [22], although a positive effect was observed in the osteoblast-like cells. In a study conducted with human CD14+ cells, it was observed that sodium silicate at 500 *μ*M caused a slight decrease in calcium phosphate resorbing ability, but no effects on cell viability were observed [24]. In another work, Si in the form of bioglass particles or released from those particles to the culture medium did not affect RAW264.7 cellular viability for concentrations < 200 *μ*M [10], in cocultures with mouse bone marrow cells; however, a decrease in osteoclast development was observed. It was observed that human osteoclasts revealed a higher activity in the presence of biomaterials containing Si compared to the corresponding biomaterial alone [23, 25]. An opposite behaviour was observed in another study, where nanograined hydroxyapatite containing Si promoted a decrease in osteoclastogenesis [26]. Thus, though there are some contradictory results and the experimental conditions (cell types, Si formulation, and dose) are very different among the published reports, the majority of them point towards an inhibitory effect of Si on osteoclastogenesis, which was corroborated in the present study.

In order to gather some insights about the intracellular mechanisms modulated by Si, cell cultures were treated with several different inhibitors of signalling pathways known to be involved in osteogenesis and osteoclastogenesis [1, 3, 6, 9]. MEK and PKC pathways appeared to be upregulated by Si in both cell cultures. In the case of hMSC, an involvement of MAPKK pathway and PGE2 production was also noted. Regarding CD14+ cell cultures, JNK was also downregulated. Osteoblastogenesis and osteoclastogenesis are very complex processes that require an intricate network of cross-talks between different intracellular signalling molecules that belong to different signalling pathways. Despite the considered cell type (osteoblast or osteoclast), the observed upregulation of MEK and PKC suggests that both pathways may be important players in Si-related bone anabolic effects. Nevertheless, it is important to note that it is the sum of all the individual contributions of signalling pathways (either those analyzed or others) that dictates the differentiation state of bone precursor cells. Also, specific effects in each cell type may account for the global effects of Si on bone tissue, as is the case of the observed upregulation of MAPKK and PGE2 production on osteoblasts and the downregulation of JNK on osteoclasts. Little information is available regarding the molecular mechanisms affected by Si in bone cells. It was previously demonstrated that Si has the ability to upregulate MEK pathway in MG63 cells [32], which is in line with the present results, although the tested concentrations were 2 and 4 mM, which are significantly higher than the naturally occurring levels in human plasma. In another study, it was demonstrated that silicified collagen scaffolds, particularly collagen scaffolds containing Si and calcium, have the ability to decrease osteoclastogenesis in a coculture model of mouse bone marrow and RAW264.7 cells, by modulating MEK and particularly p38 signalling pathways [39].

**Figure 6:**
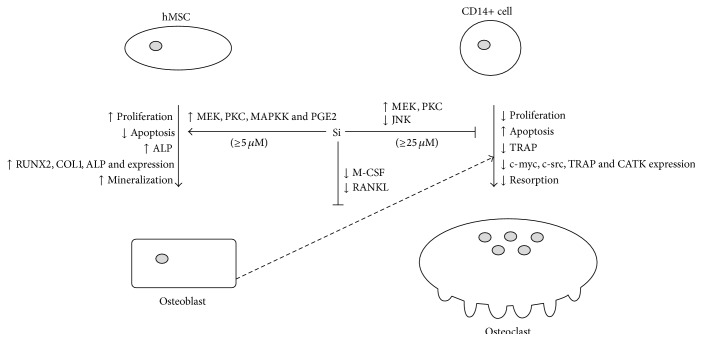
Effects of Si on human osteogenesis and osteoclastogenesis and proposed mechanisms.

Taken together, since the osteogenic effects of Si were observed in a concentration range that corresponds to the normal plasma Si levels [12, 16, 18], Si may be regarded as a permanent physiological positive modulator of bone anabolic activities. However, a maximum response was achieved at higher Si concentrations that fall within the physiological range only observed after a meal, which means that Si intake may be an additional modulation factor of bone anabolism. Also, a direct negative effect on osteoclast differentiation and function was only observed at the same higher concentrations, which suggests that this process may be regarded as an additional contribution of Si on bone anabolism, though in this case it may not represent its main physiological action of Si, at least at basal levels. Nevertheless, it is important to note that a downregulation of M-CSF and RANKL expression was also observed in the presence of low levels of Si, which suggests that Si may also exert an antiosteoclastogenic effect via osteoblastic cells [1, 2], in addition to the observed direct effects on osteoclasts. The modulation of MEK and PKC signalling pathways appeared to be particularly important for the observed cellular responses in both cell types.  Figure 6 summarizes the Si effects on bone cells and the proposed mechanisms. Thus, Si is an important natural modulator of bone metabolism, and nutrition has the ability to modulate such behaviour, which may be crucial for bone health promotion and also in bone regeneration strategies.

## Figures and Tables

**Table 1:** qPCR primer sequences of the studied genes.

Gene	Primer sequences	
*GUSB*	5′-TGCAGCGGTCTGTACTTCTG	5′-CCTTGACAGAGATCTGGTAATTCA-3′
*PSMB6*	5′-GCCGGCTACCTTACTACCTG	5′-AAACTGCACGGCCATGATA-3′
*RUNX2*	5′-GGTTAATCTCCGCAGGTCAC-3′	5′-CACTGTGCTGAAGAGGCTG-3′
*COL1*	5′-TCCGGCTCCTGCTCCTCTTA-3′	5′-ACCAGCAGGACCAGCATCTC-3′
*ALP*	5′-ACGTGGCTAAGAATGTCATC-3′	5′-CTGGTAGGCGATGTCCTTA-3′
*OC*	5′-ACACTCCTCGCCCTATTG-3′	5′-GATGTGGTCAGCCAACTC-3′
*BSP*	5′-GCATCGAAGAGTCAAAATAG-3′	5′-TTCTTCTCCATTGTCTTCTC-3′
*OP*	5′-AGGAGGAGGCAGAGCACA-3′	5′-CTGGTATGGCACAGGTGATG-3′
*M-CSF*	5′-CCTGCTGTTGTTGGTCTGTC-3′	5′-GGTACAGGCAGTTGCAATCA-3′
*RANKL*	5′-GAGCGCAGATGGATCCTAAT-3′	5′-TCCTCTCCAGACCGTAACTT-3′
*c-myc*	5′-TACCCTCTCAACGACAGCAG-3′	5′-TCTTGACATTCTCCTCGGTG-3′
*c-src*	5′-AAGCTGTTCGGAGGCTTCAA-3′	5′-TTGGAGTAGTAGGCCACCAG-3′
*TRAP*	5′-ACCATGACCACCTTGGCAATGTC-3′	5′-ATAGTGGAAGCGCAGATAGCCG-3′
*CATK*	5′-AGGTTCTGCTGCTACCTGTGGTG-3′	5′-CTTGCATCAATGGCCACAGAGAC-3′
*CA2*	5′-GGACCTGAGCACTGGCATAAGG-3′	5′-AAGGAGGCCACGAGGATCGAAG-3′
